# Alteration of NPY in hypothalamus and its correlation with leptin and ghrelin during the development of T2DM in a rat model

**DOI:** 10.1186/s40064-016-3555-9

**Published:** 2016-11-03

**Authors:** Qing-jiu Zhang, Chang-chun Yang, Song-yun Zhang, Li-hui Zhang, Jie Li

**Affiliations:** 1 Department of Neurosurgery, The Second Hospital of Hebei Medical University, Shijiazhuang, 050000 Hebei People’s Republic of China; 2 The First Department of South Storied Building, The General Hospital of Chinese People’s Armed Police Forces, Beijing, 100039 People’s Republic of China; 3 Department of Endocrinology, The Second Hospital of Hebei Medical University, No. 215 Hepingxi Road Xinhua District, Shijiazhuang, 050000 People’s Republic of China

**Keywords:** Type 2 diabetes mellitus, Insulin, Neuropeptide Y (NPY), Leptin, Ghrelin, Hypothalamus

## Abstract

**Objectives:**

This study aimed to investigate the alteration of Neuropeptide Y (NPY) in the hypothalamus and its correlation with insulin, leptin and ghrelin during the development of a rat model of type 2 diabetes mellitus.

**Methods:**

The type 2 diabetes mellitus model was developed in diet-induced obesity (DIO) rats followed by the intraperitoneal injection of low-dose streptozotocin (STZ, 25 mg/kg). At four time points during the development of type 2 diabetes mellitus in rats, the fasting serum insulin, leptin, and plasma ghrelin were measured and the hypothalamic neuropeptide Y (NPY) content and mRNA expression were detected in the rats, which were divided into 4 groups: normal control (NC), DIO_4W_, DIO_8W_, and T2DM; the mRNA expression of OB-Rb, and GSH-R1a in the hypothalamus were also assayed.

**Results:**

During the development of the type 2 diabetes mellitus rat model, both the fasting serum levels of insulin and leptin (ng/ml) elevated significantly and the fasting plasma ghrelin concentration decreased the hypothalamic NPY (pg/mg) content significantly. NPY mRNA increased significantly in a time-dependent fashion while both the OB-Rb and the GHS-R1a mRNA of the hypothalamus decreased significantly. Hypothalamic NPY concentration was positively correlated with the changes in serum insulin and leptin and negatively correlated with plasma ghrelin.

**Conclusions:**

During the development of the type 2 diabetes mellitus rat model, the hypothalamic NPY content and NPY mRNA expression increased in a time-dependent manner, which was positively correlated with the changes of the serum insulin and leptin and negatively correlated with the plasma ghrelin.

## Background

Type 2 diabetes mellitus (T2DM) is a metabolic disorder that is caused by the interaction of genetic and environmental factors. Neuropeptide Y (NPY) is a potent appetite-stimulating hormone that mainly comes from the hypothalamic arcuate nucleus (ARC) (Sousa-Ferreira et al. 2011), which plays a pivotal role in regulating of feeding behavior and energy consumption. Pathophysiologically, type 2 diabetes mellitus is characterized by hyperinsulinemia and insulin resistance. Insulin can act locally to inhibit the synthesis and release of hypothalamic NPY (Maejima et al. 2011). Ghrelin is predominantly produced by the gastric mucosa, which stimulates the appetite and induces a positive energy balance leading to body weight gain (Kirsz and Zieba 2011). Leptin is secreted principally by the adipocytes of the white adipose tissue and can curb the appetite and reduce the formulation and accumulation of fat, both of which are closely associated with the occurrence of type 2 diabetes mellitus (Zhang et al. 2013). Moreover, NPY-positive cells located in hypothalamic ARC are the site of action for both ghrelin and leptin. Ghrelin stimulates the synthesis and secretion of NPY, whereas leptin inhibits it (Druce and Bloom 2012). During the development of the type 2 diabetes mellitus rat model, the changes in hypothalamic NPY contents and its correlation with the insulin, leptin, and ghrelin levels, as well as the potential mechanism underlying their interactions have not yet been reported. This study aimed to investigate the changes of hypothalamic NPY levels and the main hormones that regulate the synthesis and release of hypothalamic NPY, including insulin, leptin, and ghrelin, and may provide additional insights into the study of the pathogenesis of type 2 diabetes.

## Methods

### Animal grouping and model preparation

Seventy male Sprague–Dawley rats (6 weeks of age) with a mean body weight of 200 g were provided by the Laboratory Animal Center of Hebei Medical University, China (Certificate Number: DK0408-0089). All rats had free access to food and water and were given 1 week to adapt to the new environment before any procedures was initiated. Of them, 10 rats assigned to the control group were fed with standard laboratory chow. The remaining 60 rats were assigned to the experimental group and fed a high-fat (HF) diet (10% more lard and 2% more cholesterol than the standard laboratory chow). After being fed a high-fat diet for 4 weeks, the rats in the experimental group were then subdivided into 2 groups according to body weight: 31 rats with a body weight of more than 2 standard deviations (g) above the mean weight of the controls were classified as the diet-induced obese (DIO) rats and selected for further study, whereas the 29 rats with body weights below this threshold were excluded. Ten of the 31 (DIO rats were killed and their blood serum and brain tissue were harvested, and this group was set as the DIO_4W_ group. The remaining 21 DIO rats were allowed to continue eat a high-fat diet for another 4 weeks, and then 10 rats were killed to collect blood and brain tissue samples at the end of the feeding trial (at the eighth week), and this group is referred to as the DIO_8W_ group. The remaining 11 rats were intraperitoneally injected with STZ (25 mg/kg) for 10 days. At the end of the STZ treatment, all 11 rats had fasting blood glucose (FBG) more than 3 standard deviations (g) above the mean levels of the controls (7.8 mmol/l) were classified into T2DM group. Therefore, type 2 diabetes mellitus models were successfully established in all of the 11 rats. Thus, the experimental animals were divided into 4 groups: control, DIO_4W_, DIO_8W_p, and T2DM groups. We used glucose clamp technology and the insulin sensitivity index (ISI) to evaluate insulin sensitivity in each group.

### Determination of general physical characteristics

General physical characteristics of rats, including appetite, behavior, hair, and excrement, were recorded every day. Body length and body weight were measured weekly and Lee’s index [Lee’s index = (weight of 1/3 × 1000)/body length (cm)] employed as a predictor of obesity in the MSG-rodents, was calculated. The fasting blood glucose levels were monitored. All fat located within the abdominal cavity was removed and their wet weight was determined afterwards. The serum total cholesterol and triglycerides were determined using a BeckmanX-20 automatic biochemical analyzer.

### Biochemical analysis

The rats were anesthetized with an intraperitoneal injection of 2.5% sodium pentobarbital (50 mg/kg). The 4 ml samples of blood were taken by heart puncture at the time of death (2 ml with anti-coagulant treatment, and 2 ml without coagulant treatment). Blood samples were centrifuged at 1600 rpm at 4 °C for 15 min, and serum (plasma) was stored at −70 °C for further assays. Serum levels of insulin and leptin were determined by radioimmunoassay using a commercial kit (intra-assay *CV* < 10%, inter-assay *CV* < 15%) from Beijing Hi-Tech Atomic Technology, Inc. (Beijing, China). Samples within a given matched set were assayed in the same batch by laboratory personnel who were blinded to the case or control status of the serum sample using a FJ-2021 γ radioactivity counter. In addition, plasma ghrelin concentrations were measured with a MK-Ascent microplate reader using enzyme-linked immunosorbent assay (ELISA) kit (Phoenix Pharmaceuticals, Inc., Burlingame, CA, USA).

### Hypothalamic NPY determination

The NPY levels in the hypothalamus were measured with specific radio immunological assays. The rats were killed and intracardially perfused with normal saline before decapitation. Subsequently, the brain tissues were boiled in 0.2 mol/l of acetic acid solution for 10 min to inactivate the enzyme. The hypothalamus was dissected and homogenized following an additional 1 ml of the acetic acid solution. After homogenization, the solution was centrifuged and the resulting supernatant was collected for further analysis. The above mentioned procedures were performed in accordance with the kit’s instructions. The kits were obtained from Shanghai Ruiqi Biological Technology Co. Ltd.(Shanghai, China).

### Determination of NPY mRNA, OB-Rb mRNA, and GHS-R1a mRNA

Total RNA was extracted from hypothalamic tissue using the TRIzol reagent (Invitrogen, Carlsbad, CA, USA) in 100 mg of tissue according to the manual. The tissues were re-dissolved in each tube with 20 μl of 0.1% DEPC-treated water. The RNA preparations, which had ratio of OD_260_/OD_280_ in the range of 1.8–2.0 determined by 756 UV–VIS Spectrophotometer, were regarded as pure and used for reverse transcription. Reverse transcription was performed with 2 µg of isolated total RNA at 42 °C using reverse transcription kit (MBI Fermentas, USA). Semi-quantitative polymerase chain reaction (PCR) amplification was employed to investigate the tissue distribution of NPY mRNA, OB-Rb mRNA, and GHS-R1a mRNA in the hypothalamic tissue. For normalization, β-actin levels were used as an internal control. PCR in the exponential amplified stage with 35 cycles of amplification was selected for NPY, OB-Rb, GHS-R1a and β-actin. The primers of NPY, GHS-R1a, OB-Rb and β-actin were all synthesized by Shanghai Biological Engineering Co., Ltd. The detailed thermal cycling conditions for PCR are shown in Table 1. Finally, RT-PCR products were separated by electrophoresis and differential display of RT-PCR gels were analyzed for quantification using a UVP imaging system. The relative mRNA expression of the target genes was calculated by the ratio of the OD value of the target band to that of the internal control band.

**Table 1:** Primer sequences and PCR thermal cycle parameters

Target gene	Primer sequence	PCR thermal cycle parameters
NPY (386 bp)	5′CAGAGCACCAGAAAGCCCAGCAG 3′ 5′ CCAACATCGAAGGGAGCATTGAA 3′	95 °C 10 min, 95 °C 5 s, 57 °C 10 s, 72 °C 30 s (35 cycles), 72 °C extended 5 min
GHS-R1a (313 bp)	5′GAGATCGCTCAGATCAGCCAGTAC3′ 5′TAATCCCCA AACTGAGGTTCTGC3′	94 °C 5 min, 94 °C 45 s, 53 °C 40 s, 72 °C 45 s (35 cycles), 72 °C extended 5 min
OB-Rb (116 bp)	5′ GCAGCTATGGTCTCACTTCTTTTG 3′ 5′ GGT TCC CTG GGT GCT CTG A 3′	94 °C 5 min, 94 °C 45 s, 52 °C 40 s,
β-actin (409 bp)	5′ GCC ATG TAC GTA GCC ATC CA 3′ 5′ GAA CCG CTC ATT GCC GAT AG 3′	72 °C 45 s (35 cycles), 72 °C extended 5 min

### Statistical analysis

Statistical analysis was performed with SPSS software (version 10.0). Central tendency and dispersion tendency were expressed as the means and standard deviation. ANOVA was used to test the differences among groups. The *t* test was employed for pair wise comparison, and linear regression/correlation analysis was performed to evaluate the interrelationship between parameters. The correlation analysis was carried out in 2 stages. In the first stage, each explanatory variable was tested using the Pearson coefficient. Then, the variables that reached a significant association at the 5% level were included in a multiple linear regression model. A *P* value of less than 0.05 was accepted as significant for all analyses in the study.

## Results

### General physical characteristics

The rats in the control group had lustrous hair and were lively, while the rats in the DIO group showed mild physical and mental sluggishness movement slowness, lower and softer fecal excretion as well as chunky and obese figures as compared with the control group. Further, in the T2DM group, the rats displayed obvious mental sluggishness and increased frequency of urination, drinking, and eating (Table 2). The body weight, length and Lee’s index and insulin resistance index of rats in each group are listed in Table 3. During the development of the type 2 diabetes mellitus rat model, the body weight and Lee’s index of the rats displayed an upward trend (*P* < 0.01). The GIR and ISI displayed a downward trend (*P* < 0.01). The body weight and Lee’s index of the DIO_4W_, DIO_8W_, and T2DM groups were increased by 55.6, 105.4 and 109.8%, respectively, while the corresponding increases in the control group were 3.94, 6.63 and 6.90%, respectively. The GIR of the DIO4 W, DIO8 W and T2DM groups were decreased by 80.15, 60.74 and 49.12%, respectively, compared with control group. The ISI were decreased by 3.55, 9.77 and 25.33%, respectively. The extent of elevation in the T2DM group was lower than those in the DIO_4W_ and DIO_8W_ groups.

**Table 2:** The characteristics for each group at the end of experiment ($\bar{x}\; \pm \;s$)

Groups	n	Water intake (ml/day)	Food intake (g/day)	Urine volume (ml/day)
Control	10	24.00 ± 1.52	16.12 ± 0.79	20.12 ± 1.17
DIO_4W_	10	35.29 ± 2.88*	19.88 ± 0.72*	33.44 ± 1.57*
DIO_8W_	10	68.37 ± 5.70*	24.43 ± 1.06*	59.72 ± 6.36*
T2DM	11	186.13 ± 4.37*	28.90 ± 0.75*^△^	144.12 ± 7.00*

* *P* < 0.01 when compared with the controls

^△^ *P* < 0.01 as compared with the DIO4W and DIO8W groups

**Table 3:** Changes in body weight, length, and Lee’s index ($\bar{x}\; \pm \;s$)

Group	n	Body weight (g)	Body length (cm)	Lee's index	GIR (mg/kg/min)	ISI
Control	15	205.23 ± 10.05	18.38 ± 0.67	320.92 ± 7.35	10.22 ± 0.04	−4.50 ± 0.13
DIO_4W_	15	319.29 ± 11.89*	20.49 ± 0.86	333.57 ± 11.79*	8.19 ± 0.09*	−4.66 ± 0.08*
DIO_8W_	15	421.45 ± 14.09*	21.91 ± 0.97*	342.19 ± 14.10*	6.99 ± 0.10*	−4.94 ± 0.12*
DM	15	430.48 ± 15.13*	22.01 ± 1.05*	343.06 ± 7.35*	5.02 ± 0.06*	−5.64 ± 0.10*

* P < 0.01 when compared with the controls

### Adipose tissue and blood lipids

During the development of the T2DM model, the wet weight of the adipose tissue in the rats in the experiment groups was raised in a time-dependent manner, which increased respectively by 239, 366 and 418%, respectively, in rats of the DIO_4W_, DIO_8W_ and T2DM groups compared with the control group (*P* < 0.01).

**Fig. 1:**
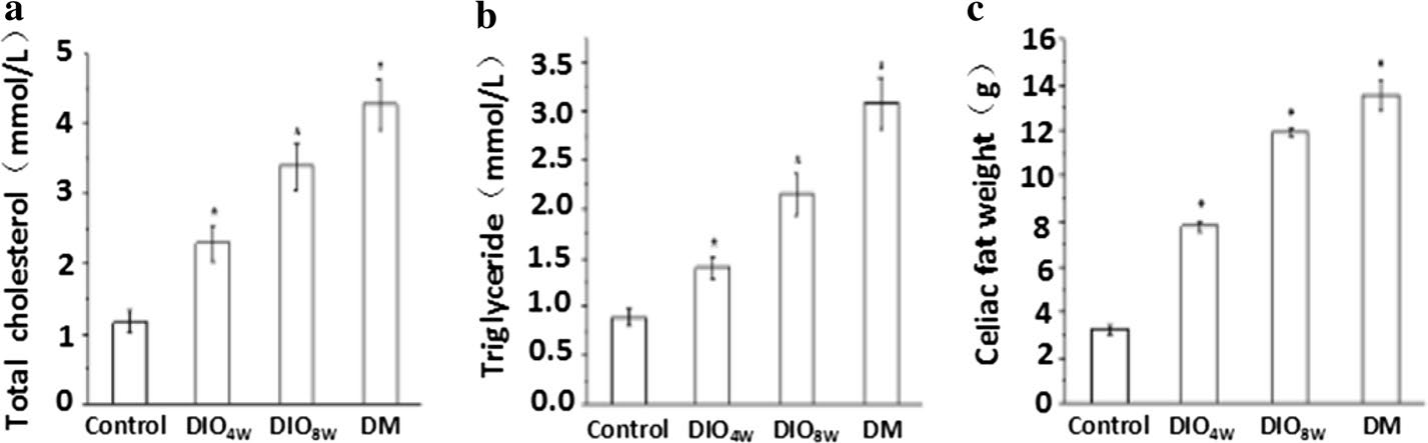
Changes in blood lipids and celiac fat weight

During the establishment of the type 2 diabetes mellitus rat model, the total serum cholesterol and triglyceride of the rats demonstrated an increasing trend (*P* < 0.01). As compared with the controls, the serum cholesterol levels were elevated by 196, 289 and 365% in the DIO_4W_, DIO_8W_ and T2DM groups, respectively, and the serum cholesterol contents were increased by 158, 242, and 347%, respectively (Fig. 1).

### Fasting blood glucose, serum insulin, serum leptin, and plasma ghrelin levels

**Fig. 2:**
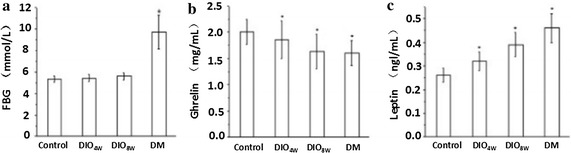
Fasting blood glucose, serum insulin and leptin, and plasma ghrelin levels

The fasting blood glucose levels in the DIO_4W_ and DIO_8W_ groups were similar to that of the control group (*P* > 0.05).Nevertheless, the fasting blood glucose levels in the T2DM group was significantly higher than that of the controls (9.71 ± 1.56 mmol/l) (*P* < 0.01). The concentrations of fasting serum insulin in the DIO_4W_ and DIO_8W_ groups were elevated in a time-dependent pattern as compared with the control group (*P* < 0.01), whereas lower fasting insulin levels were found in the T2DM group compared with that in the DIO_8W_ group (*P* < 0.01) (Table 4; Fig. 2).

**Table 4:** Fasting blood glucose, serum insulin and leptin and plasma ghrelin levels ($\bar{x}\; \pm \;s$)

Groups	n	FBG (mmol/l)	Insulin (IU/ml)	Leptin (ng/ml)	Ghrelin (ng/ml)
Control	10	5.35 ± 0.29	15.23 ± 1.65	0.26 ± 0.03	2.01 ± 0.24
DIO_4W_	10	5.42 ± 0.33	21.36 ± 1.79*	0.32 ± 0.04*	1.86 ± 0.36*
DIO_8W_	10	5.59 ± 0.30	28.28 ± 1.89*	0.39 ± 0.05*	1.63 ± 0.33*
T2DM	11	9.69 ± 1.56*	23.34 ± 1.59*^△^	0.46 ± 0.06*	1.60 ± 0.24*

* *P* < 0.01 when compared with the controls

^△^ *P* < 0.01 as compared with the DIO_8W_ group

Increasing levels of the fasting serum leptin was observed from the control group to the T2DM group (*P* < 0.01). The concentrations of fasting plasma ghrelin in the DIO_4W_ and DIO_8W_ groups decreased in a time-dependent fashion as compared with the control group (*P* < 0.01); however, no significant difference was found between the DIO_8W_ and the T2DM groups (*P* > 0.05).

### Hypothalamic NPY contents and NPY mRNA expression

#### Hypothalamic NPY content

The hypothalamus NPY contents were 133.4 ± 17.1 (pg/ml) in the normal control group, and were 160.9 ± 21.7, 194.8 ± 37.0, and 251.1 ± 40.9 (pg/ml), respectively in the DIO_4W_, DIO_8W_, and T2DM group. With the development of the type 2 diabetes mellitus rat model, the hypothalamic NPY content increased in a time-dependent manner in the rats (*P* < 0.01). Compared with the normal control group, the hypothalamic NPY levels increased by 20.6, 46.0, and 88.2%, respectively, in the DIO_4W_, DIO_8W_, and T2DM groups (the differences between the 2 adjacent groups were 20.6, 21.1, and 28.9%).

#### Hypothalamic NPY mRNA expression

**Fig. 3:**
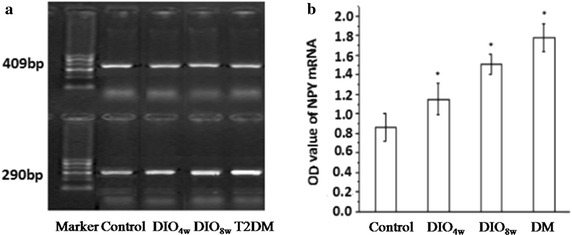
RT-PCR analysis of hypothalamic NPY mRNA expression. NPY mRNA were detected during the development of the T2DM in a rat model, based on which the rats were divided into 4 groups: normal control (NC), DIO4 W (fed a high-fat diet for 4 weeks), DIO8 W (fed a high-fat diet for 8 weeks) and T2DM group (fed a high-fat diet for 8 weeks + STZ intraperitoneal injection). NPY mRNA increased significantly in a time-dependent fashion: The OD values of NPY mRNA expression for the DIO4 W, DIO8 W, and T2DM groups were significantly higher than that of the control group (*P* < 0.01). *P < 0.05 vs. NC group

With the development of the type 2 diabetes mellitus model in rats, the expression of hypothalamic NPY mRNA showed a time-dependent increase. The OD values of NPY mRNA expression for the DIO_4W_, DIO_8W_ and T2DM groups were 1.15 ± 0.16, 1.51 ± 0.14 and 1.78 ± 0.14, respectively, all of which were significantly higher than that of the control group (0.86 ± 0.14, *P* < 0.01) (Fig. 3).

### Hypothalamic GHS-R1a and OB-Rb mRNA

**Fig. 4:**
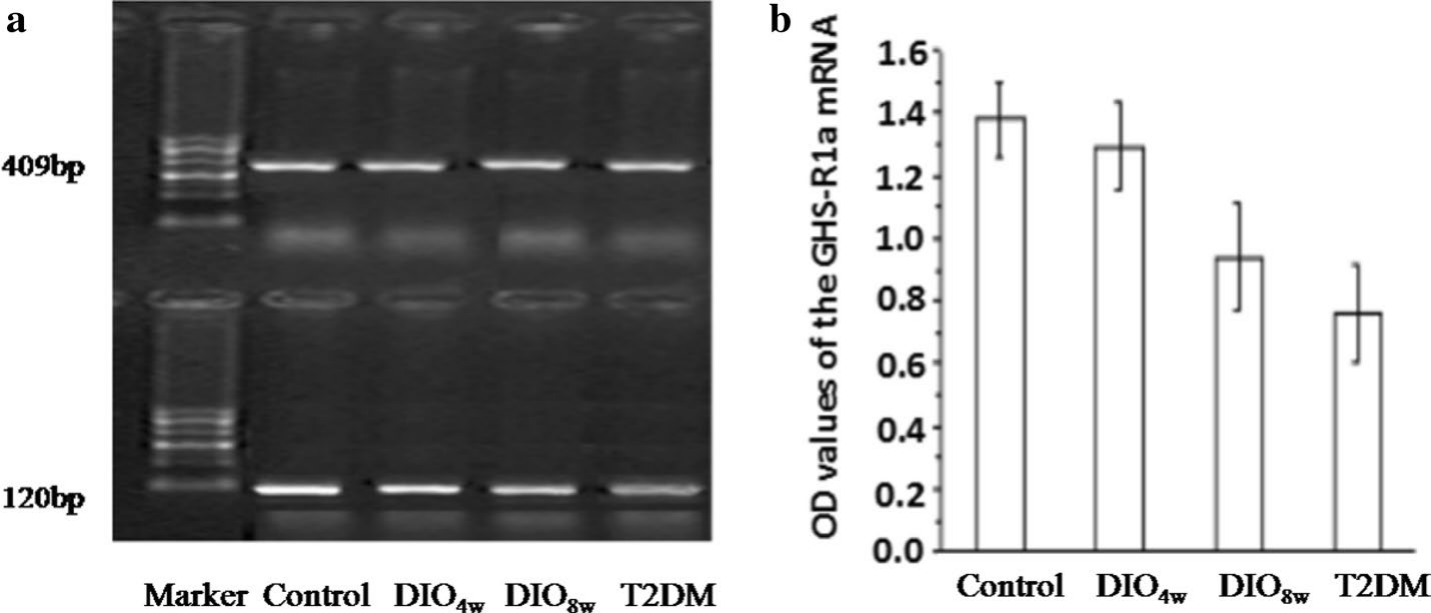
RT-PCR analysis of hypothalamic GHS-R1a mRNA expression. The expression of GHS-R1a mRNA was detected respectively in normal control group (NC), DIO4 W group (feed on high fat diet for 4 weeks), DIO8 W group (fed a high-fat diet for 8 weeks), and T2DM groups (fed a high-fat diet for 8 weeks +STZ intraperitoneal injection), respectively. The OD values of the GHS-R1a mRNA expression in hypothalamus in the control, DIO4 W, DIO8 W, and T2DM groups were 1.38 ± 0.12, 1.29 ± 0.14, 0.94 ± 0.17 and 0.76 ± 0.16 respectively, which showing a downward trend (*P* < 0.01). *P < 0.05 vs. NC group

**Fig. 5:**
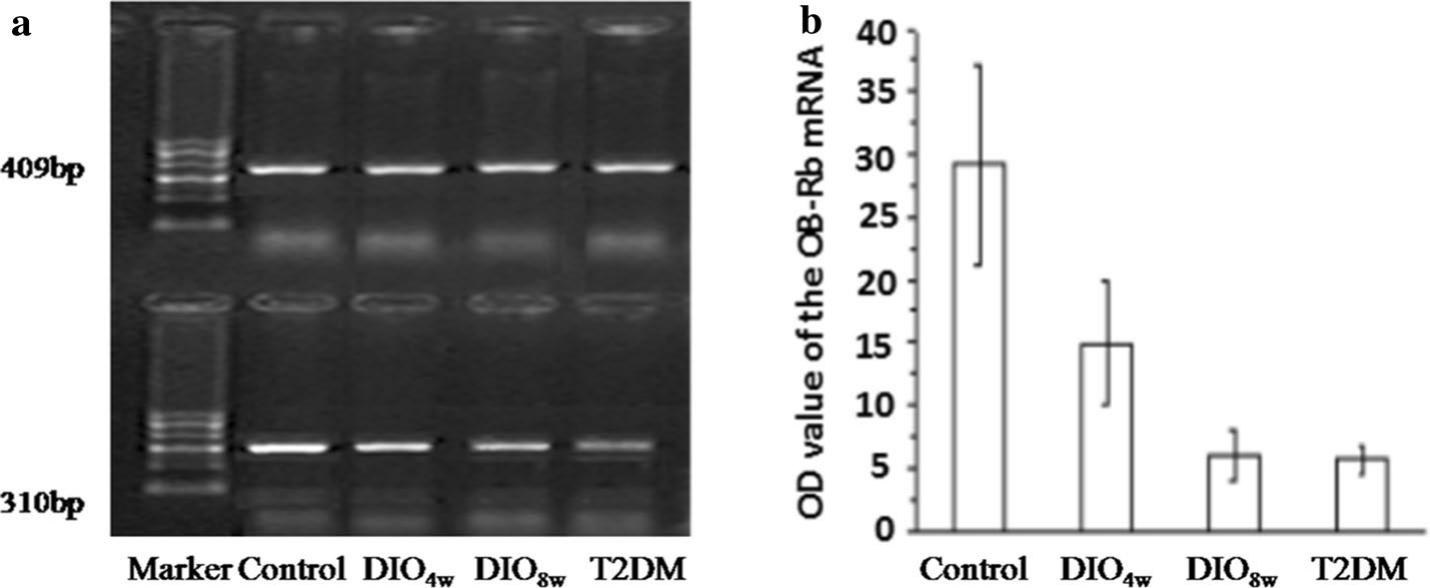
RT-PCR analysis of hypothalamic OB-Rb mRNA expression. The OD values of the OB-Rb mRNA were 29.15 ± 7.96, 14.89 ± 4.98, 5.96 ± 2.12 and 5.61 ± 1.13 in the normal control (NC), DIO4 W (fed a high-fat diet for 4 weeks), DIO8 W group (fed a high-fat diet for 8 weeks), and T2DM groups (fed a high-fat diet for 8 weeks + STZ intraperitoneal injection), and also showed a downward trend (*P* < 0.01). *P < 0.05 vs. NC group

The OD values of the GHS-R1a mRNA were 1.38 ± 0.12, 1.29 ± 0.14, 0.94 ± 0.17 and 0.76 ± 0.16, and also showed a downward trend (*P* < 0.01) (Fig. 4). The OD values of the OB-Rb mRNA expression in hypothalamus in the control, DIO_4W_, DIO_8W_, and T2DM groups were 29.15 ± 7.96, 14.89 ± 4.98, 5.96 ± 2.12 and 5.61 ± 1.13, respectively, which showed a downward trend (*P* < 0.01) (Fig. 5).

### Correlation analysis

The contents of hypothalamic NPY in all of the groups were positively associated with the fasting serum insulin (*r* = 0.892, *r* ^*2*^ = 0.793, *P* < 0.01) and leptin levels (*r* = 0.951, *r* ^*2*^ = 0.905, *P* < 0.01). Nevertheless, it was inversely correlated with the fasting plasma ghrelin levels (*r* = −0.888, *r* ^*2*^ = 0.789, *P* < 0.01).

## Discussion

In the present study, the conventional type 2 diabetes mellitus rat model protocol was modified (Zhang et al. 2010) by administering intraperitoneal injection of small doses of STZ (30 mg/kg) to the DIO rats instead of the high-fat diet rats. The modified procedure may produce a type 2 diabetes mellitus rat model that can better mimic the occurrence and development of human type 2 diabetes mellitus, with the existence of peripheral insulin resistance and mildly impaired pancreas function, which causses a stable insulin resistance. Therefore, this procedure is more in line with the pathogenesis of human type 2 diabetes mellitus.

Several indexes, such as body weight, Lee’s index, and fat wet weight were measured to determine the obesity and fat accumulation of the laboratory animals. The results showed that during the establishment of the type 2 diabetes mellitus rat model, all of the above mentioned indicators presented an increasing trend, which confirmed that obesity can be induced by a high-fat diet in a quite short term. However, the magnitude of weight gains in the DIO_8W_ group was lower than that in the DIO_4W_ group; part of the reason for this phenomenon may lie in the occurrence of diabetes.

This study confirmed that during the development of a type 2 diabetes mellitus rat model (from the control group to the T2DM group), the expression of the hypothalamic neuropeptide Y increased in a time-dependent manner as the body weight and body fat elevated, which is consistent with the reports of the previous studies (Polkowska et al. 2012). Therefore, neuropeptide Y serves as the most potent appetite stimulator, which can reduce energy consumption and may play a key role in the onset of both obesity and type 2 diabetes mellitus (Parker and Bloom 2012).

The neuropeptide Y system is considered to be the final common pathway for appetite expression in the hypothalamus. Leptin is an appetite-suppressing peptide, while ghrelin is an appetite-stimulating one. They antagonize each other in regulating energy balance (Yang et al. 2010), and regulate appetite and eating behavior by directly signaling to the hypothalamic neuropeptide Y neurons. Moreover, insulin is another important hormone that can regulate hypothalamic neuropeptide Y secretion, and increased insulin secretion can inhibit the synthesis and release of the hypothalamic neuropeptide Y (Martins et al. 2012; Fick and Belsham 2010; Duarte et al. 2012).

The results demonstrated that serum insulin and leptin levels increased, whereas plasma ghrelin concentration decreased with the increase of body weight and body fat in the DIO_4W_ and DIO_8W_ groups when compared with the controls during the development of type 2 diabetes mellitus model in rats, which was consistent with the results of our previous research on high-fat diets and STZ-induced type 2 diabetes mellitus in rats (Briggs et al. 2010). Plasma insulin and leptin enters the central nervous system though the blood brain barrier and interacts with the specific insulin and the receptor of leptin (OB-Rb) receptors in the hypothalamic ARC, thereby suppressing the hypothalamic ARC NPY expression and curbing food intake (Laron 2009). Ghrelin binds to and activates GH secret agogue receptor type 1a (GHS-R1a), thus stimulating the NPY expression and promoting food intake (Morton and Schwartz 2011). Nevertheless, insulin resistance and leptin resistance was induced by high-fat diet in the DIO rats (Kirsz and Zieba 2011). On the one hand, it led to the abnormal transport through the blood–brain barrier of leptin and insulin, leading to decreased levels of insulin and leptin in the CNS despite of the existence of hyperinsulinemia and hyperleptinemia. On the other hand, it might result in receptor and post-receptor defects and block normal signal transduction, especially for inhibitory effect of insulin and leptin on hypothalamic ARC NPY expression, leading to elevated NPY levels in hypothalamus. Leptin is a powerful inhibitor of ghrelin (Verhulst et al. 2012), which can act directly on gastric mucosa to inhibit ghrelin release. In addition, increased insulin secretion can suppress the plasma ghrelin concentration as well (Andrews 2011). Thus, during the development of the type 2 diabetes, increasing serum concentrations of insulin and leptin, decreasing plasma ghrelin, and declining ghrelin’s stimulation on hypothalamic ARC NPY neurons were observed from the control group to the DIO_8W_ group. It has to be mentioned that the hypothalamic NPY neurons were innervated by several types of hormones, and the mechanisms by which the NPY concentrations somehow remained high need to be further explored. Generally speaking, the decrease in plasma ghrelin concentrations can be regarded as the body’s negative feedback regulation in response to the positive energy balance, by which appetite and food intake can be inhibited and further weight gain may be prevented.

Hyperleptinemia persisted from the DIO_8W_ group to the T2DM group throughout the experiment. Nevertheless, the serum insulin levels in the T2DM group were lower than that in the DIO_8W_ group, which might be attributed to the fact that STZ can directly damage the pancreas following intraperitoneal injection, leading to a consequent sharp decrease in insulin secretion (Briggs et al. 2010). Central insulin levels might decrease with the reduction in peripheral insulin levels as well, which further weakens its inhibitory effect on the NPY neurons in the hypothalamus ARC. As a result, the contents of hypothalamic NPY increased significantly (up to 28.9%). Furthermore, after completion of the type 2 diabetes mellitus model, the concentrations of fasting plasma ghrelin did not continue to decrease. It might due to the weakened inhibition influence of insulin on ghrelin following the significant reduction of serum insulin concentrations, and the increased plasma ghrelin further boosted the expression of hypothalamic NPY.

These results are consistent with those of our previous studies (Zhang et al. 2013) and suggest that both the hypothalamic OB-Rb and GHS-R1a mRNA expression were reduced with the development of the type 2 diabetes mellitus rat model. The findings suggest that the reduced expression of OB-Rb mRNA is another pivotal mechanism underlying leptin resistance in addition to the abnormal blood–brain transport of leptin. Furthermore, the decreased GHS-R1a mRNA expression is believed to be related to ghrelin resistance (Briggs et al. 2010).
